# Establishment of an orthotopic prostate cancer xenograft mouse model using microscope-guided orthotopic injection of LNCaP cells into the dorsal lobe of the mouse prostate

**DOI:** 10.1186/s12885-022-09266-0

**Published:** 2022-02-16

**Authors:** Weiyong Liu, Yunkai Zhu, Lei Ye, Yajuan Zhu, Yuhao Wang

**Affiliations:** 1 Department of Ultrasound, Division of Life Sciences and Medicine, The First Affiliated Hospital of USTC, University of Science and Technology of China, Hefei, 230036 Anhui People’s Republic of China; 2 Department of Ultrasound, Xinhua Hospital Affiliated to Shanghai Jiaotong University, 200092 Shanghai, People’s Republic of China; 3 Department of Clinical Medicine, Wannan Medical College, Wuhu, 241002 Anhui People’s Republic of China

**Keywords:** Mouse prostate anatomy, LNCaP, Orthotopic xenograft, Ultrasonography, Pathology

## Abstract

**Background:**

Orthotopic LNCaP xenograft mouse models closely mimic the progression of androgen-dependent prostate cancer in humans; however, orthotopic injection of LNCaP cells into the mouse prostate remains a challenge.

**Methods:**

Under the guidance of a stereoscopic microscope, the anatomy of the individual prostate lobes in male Balb/c athymic nude mice was investigated, and LNCaP cells were inoculated into the mouse dorsal prostate (DP) to generate orthotopic tumors that mimicked the pathophysiological process of prostate cancer in humans. Real-time ultrasound imaging was used to monitor orthotopic prostate tumorigenesis, contrast-enhanced ultrasonography (CEUS) was used to characterize tumor angiogenesis, and macroscopic and microscopic characteristics of tumors were described.

**Results:**

The DP had a trigonal bipyramid-shape and were located at the base of the seminal vesicles. After orthotopic inoculation, gray scale ultrasound imaging showed progressive changes in tumor echotexture, shape and location, and tumors tended to protrude into the bladder. After 8 weeks, the tumor take rate was 65% (*n* = 13/20 mice). On CEUS, signal intensity increased rapidly, peaked, and decreased gradually. Observations of gross specimens showed orthotopic prostate tumors were well circumscribed, round, dark brown, and soft, with a smooth outer surface and a glossy appearance. Microscopically, tumor cells were arranged in acini encircled by fibrous septa with variably thickened walls, mimicking human adenocarcinoma.

**Conclusions:**

This study describes a successful approach to establishing an orthotopic LNCaP xenograft Balb/c athymic nude mouse model. The model requires a thorough understanding of mouse prostate anatomy and proper technique. The model represents a valuable tool for the in vivo study of the biological processes involved in angiogenesis in prostate cancer and preclinical evaluations of novel anti-angiogenic therapies.

## Background

Prostate cancer is one of the most commonly diagnosed cancers in men and the second leading cause of cancer-related death worldwide [1]. Early androgen deprivation therapy is associated with favorable outcomes, but many patients eventually develop metastatic castration-resistant disease, which has a high mortality rate [2]. The mechanisms underlying the transition from hormone sensitive to castration-resistant disease remain unclear. Animal models that mimic the natural history of prostate cancer in humans are required to improve our understanding of disease progression.

The LNCaP cell line constitutes androgen receptor-positive prostate cancer cells that express prostate-specific antigen (PSA) and prostate specific membrane antigen (PSMA), a marker of disease aggressiveness [3]. Subcutaneous and orthotopic LNCaP xenograft animal models closely mimic the progression of androgen-dependent prostate cancer in humans. Compared with subcutaneous models, orthotopic models more accurately replicate an organ microenvironment that preserves original tumor cell phenotypes [4]. However, the development of reproducible orthotopic prostate cancer xenografts is more demanding than a subcutaneous procedure. The inoculation of orthotopic tumors in the prostate of nude mice is technically challenging, and the take rate of LNCaP cells is low, even with the addition of Matrigel [5].

Successful inoculation of the mouse prostate may be achieved with an open surgery approach and the assistance of a surgical microscope [6, 7]. Several orthotopic prostate cancer xenograft mouse models have been established by inoculating tumor cells into the anterior prostate (AP), as the anterior lobes are the largest and easiest to puncture [8]. More recently, there is increasing interest in inoculation into the dorsal prostate (DP), even though it is less accessible. The mouse DP is androgen sensitive and considered homologous to the human peripheral zone, while the mouse AP has less clinical significance as it is similar to the human central zone [9]. To the authors’ knowledge, there is no comprehensive protocol describing microscope-guided orthotopic injection of LNCaP cells into the mouse DP. A clear understanding of the anatomy of the mouse prostate must be established before attempting surgical orthotopic inoculation, but detailed descriptions of the individual prostate lobes in the mouse are scarce [7].

The objective of this study was to establish an orthotopic LNCaP xenograft Balb/c athymic nude mouse model as a clinically relevant animal model of prostate cancer. Under the guidance of a stereoscopic microscope, we investigated the anatomy of the individual prostate lobes in male Balb/c athymic nude mice and inoculated LNCaP cells into the mouse DP to generate orthotopic tumors that mimicked the pathophysiological process of prostate cancer in humans. Orthotopic prostate tumorigenesis was monitored with real-time ultrasound imaging. After successfully establishing the orthotopic LNCaP xenograft mouse model, contrast-enhanced ultrasonography (CEUS) was used to characterize tumor angiogenesis, and tumor histopathological and immunohistochemical characteristics were explored.

## Materials and methods

### Mouse prostate gross anatomy

22 male Balb/c athymic nude mice (5 weeks old) were purchased from Shanghai Lab Animal Research Center. Mice were acclimated for 3 weeks under pathogen-free conditions. Mice were anesthetized in an isoflurane chamber and placed in a supine position with their extremities fixed to the dissecting board. The abdominal region was disinfected, and the abdominal cavity was accessed via an incision made through the linea alba using Metzenbaum scissors. The bladder and seminal vesicles (SVs) were lifted with dissecting forceps to reveal the four lobes of the mouse prostate: AP, DP, lateral prostate (LP), and ventral prostate (VP). The prostate was viewed under a stereoscopic microscope (Zeiss Stemi 2000-C with image processing software). The AP, DP, LP, and VP were identified in the coronal, axial, cranio-lateral, and ventral views, respectively.

### Tumor cell line

The LNCaP cell line was obtained from the China Center for Type Culture Collection. Cells were cultured in DMEM/F12 (Gibco, Grand Island, NY, USA) supplemented with 10% fetal bovine serum (FBS, Gibco) and maintained in an incubator (HERACELL 150i, Thermo Scientific, Rockford, IL, USA) at 37 °C in a humidified atmosphere with 5% CO_2_. At approximately 80% confluence, cells were harvested with 0.25% trypsin/EDTA solution and washed twice in PBS. Cells (1 × 10^6^) in 20μL PBS medium were mixed with 50% Matrigel (BD Biosciences) before inoculation.

### Orthotopic injection of LNCaP cells

**Fig. 1:**
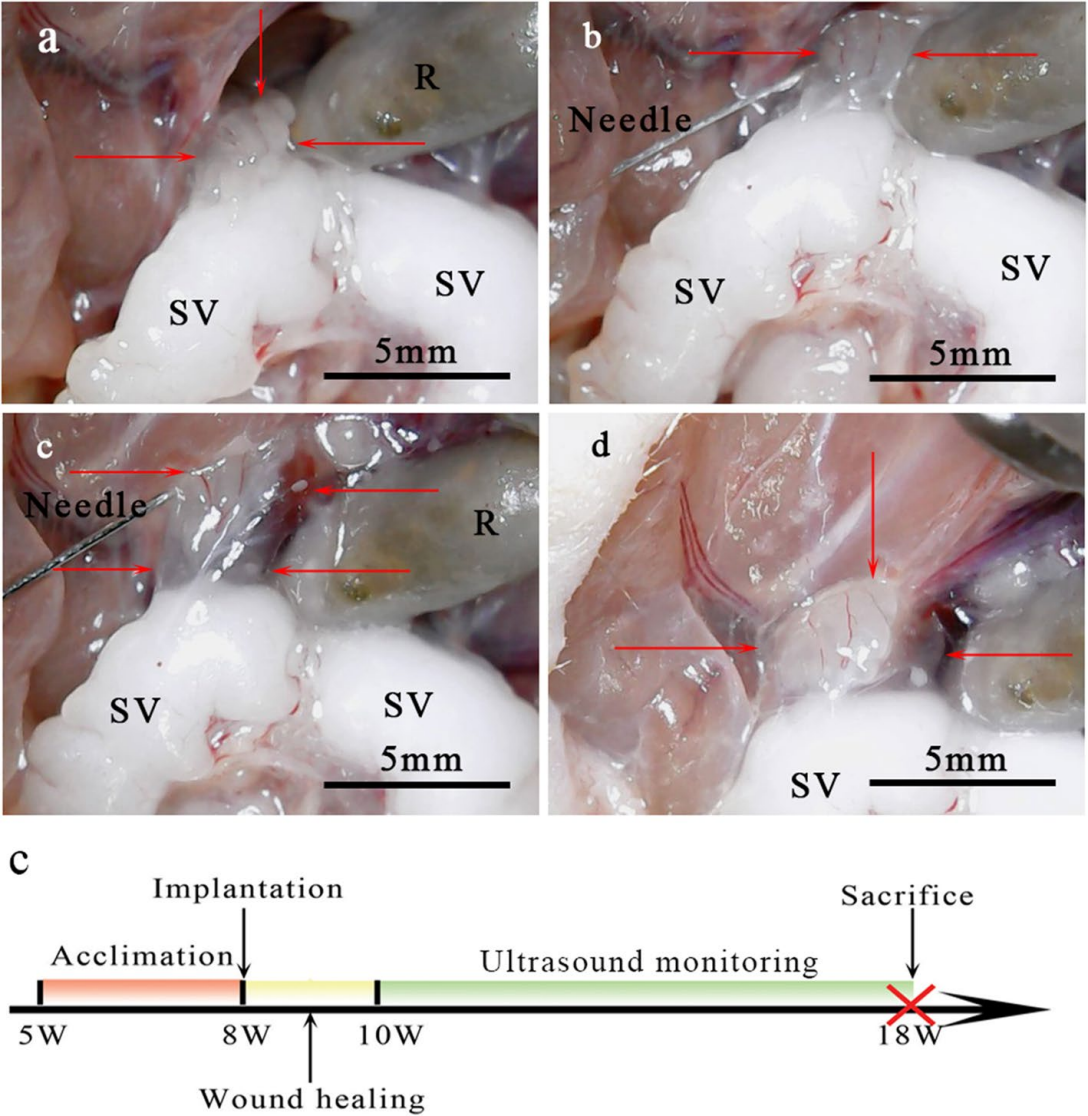
Inoculation of LNCaP cells into the right dorsal lobe (red arrow) of the prostate of male Balb/c mice and a schematic of the experimental design. **A** The trigonal bipyramidal dorsal lobe of the prostate was located. **B** A needle attached to a Hamilton syringe was inserted into the right dorsal lobe, approximately parallel to the long axis of the dorsal lobe. **C** A 20μL suspension containing 1 × 10^6^ LNCaP cells was slowly injected. **D** A small blister was observed after the needle was retracted. **E** Schematic of the experimental design. Twenty-two male Balb/c athymic nude mice (5 weeks old) were acclimated for 3 weeks, and orthotopic injection was performed at Week 8. Starting 2 weeks post-injection, tumor growth was monitored by ultrasound every 5 days. Mice were sacrificed at Week 18, and tumors were excised for histologic analysis. SV, seminal vesicle; R, rectum

22 male Balb/c athymic nude mice (5 weeks old) were acclimated for 3 weeks before experimental manipulation. LNCaP cells were grafted into the mouse left or right DP under a stereoscopic microscope shortly after the cell resuspension in Matrigel. Mice were anesthetized with 3% isoflurane in an induction chamber with flow rate of 0.8L/min and placed in a supine position. During surgery, heating pads were used to maintain body temperature. The lower abdomen was disinfected with 70% ethanol. The surgery was conducted by two operators, one immobilizing the mice and the other performing the operation. A 1.5 cm transverse incision was made in the lower midline with microscissors above the presumed location of the bladder. Approximately 100μL intraperitoneal liquid spilled and was sponged with cotton balls. The intestine was pushed upward into the abdominal cavity using a sterile cotton swab. The SV was pulled outside the abdominal cavity with micro-tweezers. The SV was moved posteriorly and stabilized with a cotton ball to expose the DP. The trigonal bipyramidal DP was located, and the needle of a Hamilton syringe (sharp 10–12° 33G beveled needle) was inserted into one side of the DP at an angle of approximately 15° to the long axis of the DP. A 20μL suspension containing 1 × 10^6^ LNCaP cells was prepared individually, and was slowly injected until the DP resembled a small blister (Fig. 1). The needle was retracted, and a cotton ball was placed over the injection site for about 1 ~ 2 s to prevent bleeding, minimize leakage, and solidify the Matrigel within the lobe. The prostate and SV were returned to the peritoneum, and the abdominal wall and skin layer were sutured with a 5–0 braided absorbable suture (Covidien). The wound was disinfected with 70% ethanol. Mice were observed until recovery (approximately 1 h). The wound healed within 2 weeks. Starting 2 weeks post-injection, tumor growth was monitored by ultrasound (US) every 5 days.

### Tumor surveillance by gray-scale US

US imaging was performed using a portable sonography device (GE LOGIQ BOOK-XP, MI 0.7, Tis 0.9, Frequency 9 ~ 11 MHz, depth 2.0 cm). Prewarmed US coupling gel was applied to the abdominal wall before imaging. 2D images of the mouse prostate and adjacent anatomy were acquired using a linear-array transducer (8L-RS) in B-mode with a center frequency of 11-MHz. The probe was positioned perpendicular to the abdominal wall in a sagittal orientation, and the bladder was identified as a round anechoic structure. The probe was moved until an echogenic tumor was identified on the dorsal side superior to the base of the bladder. Images of the bladder and tumor were acquired. Maximum sagittal length and height of the tumor was measured in the longitudinal plane. The probe was rotated to view the tumor in the transverse plane, and the width of the tumor was measured. Tumor volume was calculated by 0.52 × maximum length × height × width.

### CEUS and Time-Intensity Curves (TIC) analysis

CEUS was performed using a LOGIQ E9 system (GE Healthcare, Milwaukee, Wis., USA) with a multiple frequency linear probe (ML 6 ~ 15 MHz) prior to sacrifice. Mice were anesthetized and were examined in the supine position. The focal depth was 1 cm, and the mechanical index was 0.08 to 0.10. The tumor was visualized in the sagittal plane, and the recommended dose (100μL) of SonoVue® (Bracco, Italy) was administered as a bolus with a 1 mL syringe through the retro-orbital venous sinus. Raw data generated for 120 s were saved, and imaging datasets were analyzed with quantification software (VueBox ™). A region of interest (ROI) was manually contoured to cover the whole tumor in the longitudinal plane. A time-intensity curve (TIC) was generated based on the signal intensities in the individual ROIs.

### T_2_-weighted MRI

Magnetic resonance imaging (MRI) was performed using a 3.0 T MR system (Ingenia, Philips Medical Systems, Netherlands) with a dedicated 8-channel receive coil with a 5.0 cm inner diameter (Chenguang Medical Technologies Co., Shanghai, China) on Day 35 and Day 70 after orthotopic injection of LNCaP cells. T_2_-weighted spin-echo sequences were used to image the tumors. Transverse imaging (repetition time/echo time [TR/TE], 6.0 s/68 ms; flip angle, 90°; matrix, 64 × 64; field of view, 50 × 50 mm; slice thickness, 1.0 mm; slice gap, 0 mm; 2 signal averages) and coronal imaging (TR/TE, 3.0 s/80 ms; flip angle, 90°; matrix, 64 × 64; field of view, 100 × 100 mm; slice thickness, 1.0 mm; slice gap, 1 mm; 2 signal averages) were performed. Twenty coronal slices with a thickness of 500 mm each and an in-plane resolution of 100 mm × 100 mm were acquired with a multi-slice, multi-echo sequence. The scan time was 13 min per data set. Orthotopic tumor volume was calculated as 0.52 × width × length × height.

### Serum PSA, histological and immunohistochemistry

Blood samples were collected from the retroorbital sinus before sacrifice. PSA levels were analyzed using an Electro-ChemiLuminescence ImmunoAssay (ECLIA). Orthotopic tumors were removed, fixed in 10% neutral-buffered formalin, and embedded in paraffin. Serial Sects. (4 μm thick) were cut on a microtome and mounted on glass slides. Sections were deparaffinized in Histoclear (National Diagnostic, Atlanta, GA) and hydrated in a graded series of alcohols and running tap water. Histopathology was performed using standard hematoxylin and eosin staining. Immunohistochemical staining was performed with a Vectastain ABC Elite kit (Vector Laboratories, Inc., Burlingame, CA). Briefly, sections were deparaffinized and hydrated, and endogenous peroxidase activity was blocked with 0.3% hydrogen peroxide in methanol for 20 min. Antigen retrieval was performed using the Antigen Unmasking Solution (Vector Laboratories, Inc.). Sections were cooled and rinsed in PBS, incubated in blocking solution for at least 30 min at room temperature, and incubated with primary antibodies for CD31, VEGF-A, Ki-67 (1:300), androgen receptor (AR), PSA, p63 (1:300), or P504S overnight at 4 °C. Sections were incubated with the appropriate biotinylated secondary anti-goat, anti-mouse, or anti-rat immunoglobulin for 30 min at room temperature. The antigen–antibody reaction was visualized using 3,3’-diaminobenzidine tetrahydrochloride as a substrate. Sections were examined by light microscopy.

### Statistical analysis

Statistical analyses were performed with MedCalc software (Version 12.3.0.0, Mariakerke, Belgium). Pearson’s correlation coefficient was used to compare tumor volumes measured on MRI and US on Day 35 and Day 70. Differences in the measurement techniques were plotted using the Bland–Altman method *p* < 0.05 was considered statistically significant.

## Results and discussion

### Gross view of individual lobes

**Fig. 2:**
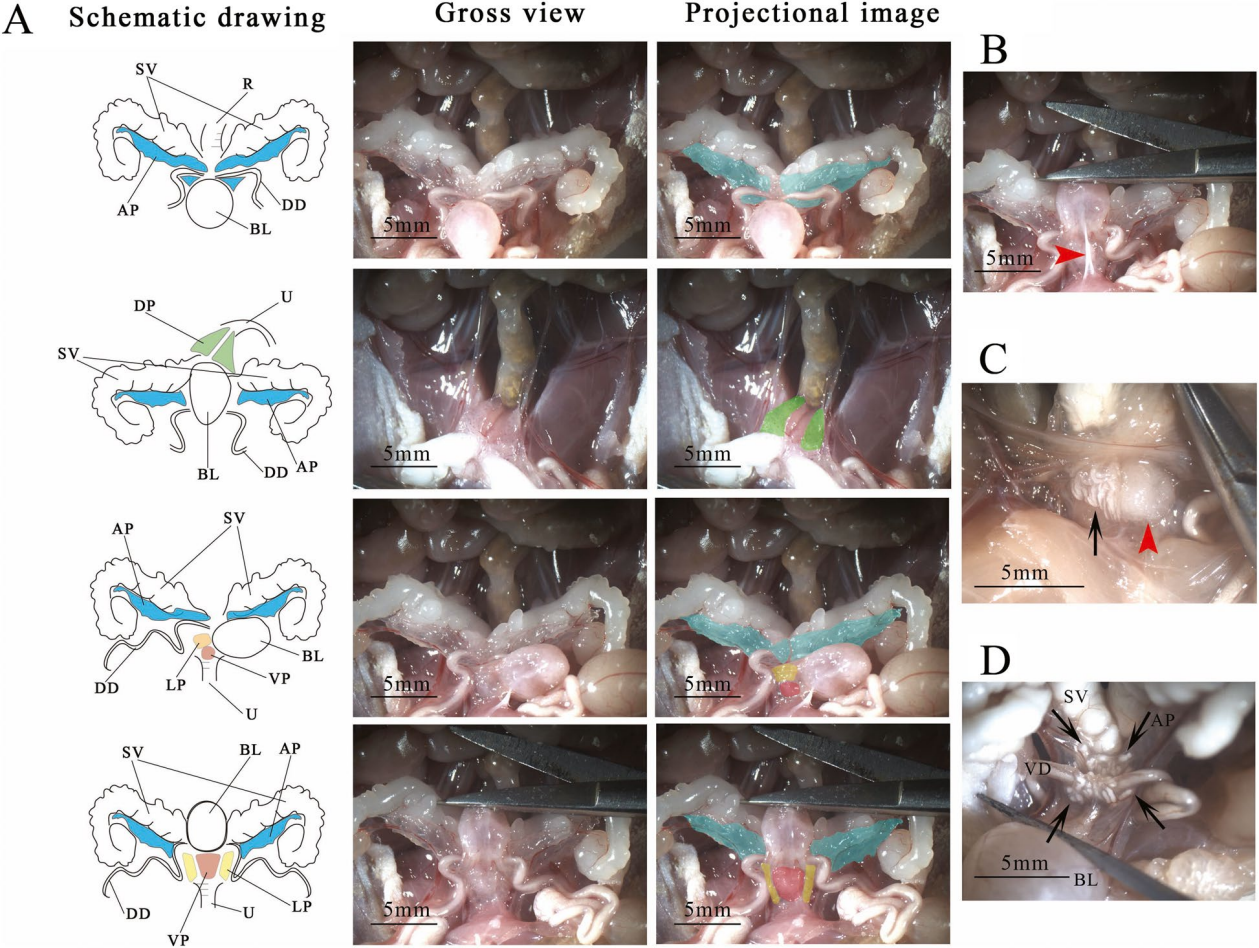
Gross view of the individual lobes of the mouse prostate. **a** Location of the VP, LP, AP, and DP. **b** The puboprostatic ligament was located anterior to the VP, and ran medially in the sagittal plane (arrow head). **c** The LP (black arrow) was adjacent to the VP (arrow head) and partially wrapped around the urethra. **d** The LP (black arrow) encircled the VD at the insertion site of the prostate. VP, ventral prostate lobe; LP, lateral prostate lobe; AP, anterior prostate lobe; DP, dorsal prostate lobes; VD, vas deferens; SV, Seminal vesicle; BL, bladder; R, rectum

The four lobes of the mouse prostate were easily visualized using a stereoscopic microscope. The AP were translucent and bilaterally attached to the lesser curvature of the SV. The DP, LP, and VP were arranged circumferentially around the urethra. The DP had a trigonal bipyramidal shape and were located at the base of the SV. The DP were lateral to the urethra on the cranio-lateral view. The bilateral LP appeared as cerebriform structures with a slightly inverted trapezium-like shape. The LP were anterior and adjacent to the VP, extended superiorly to the base of SV, and were partially contiguous with the medial aspect of the AP. The LP encircled the vas deferens (VD) at the insertion site of the prostate. The VP was round and white and appeared as a cotton ball anterior to the urethra and below the bladder. A neurovascular bundle located on the posterolateral aspect of the prostate ran parallel to the urethra and inserted obliquely into the dorsal aspect of the LP and the ventral aspect of the DP. The puboprostatic ligament was a broad, tough structure just anterior to the VP that ran medially in the sagittal plane (Fig. 2).

### Gray-scale ultrasound

After orthotopic inoculation, two mice died within 2 days; autopsy revealed bowel obstruction. Twenty Balb/c mice had no postoperative complications.

On gray-scale US, the mouse prostate had ill-defined margins and was difficult to distinguish from adjacent structures located at the base of the bladder. However, orthotopic prostate tumors were detected as early as Day 15–18 after inoculation and were easily discerned from surrounding tissues. Tumors showed progressive changes in echotexture, shape and location, which were confirmed on autopsy. At early timepoints, tumors appeared as hypoechoic, well-defined, rounded structures underneath the bladder. On Day 30 after inoculation, tumors protruded into the bladder. At later timepoints, tumors became oval-shaped and isoechoic and had a tendency to push the bladder anteriorly in the abdominal cavity.

**Fig. 3:**
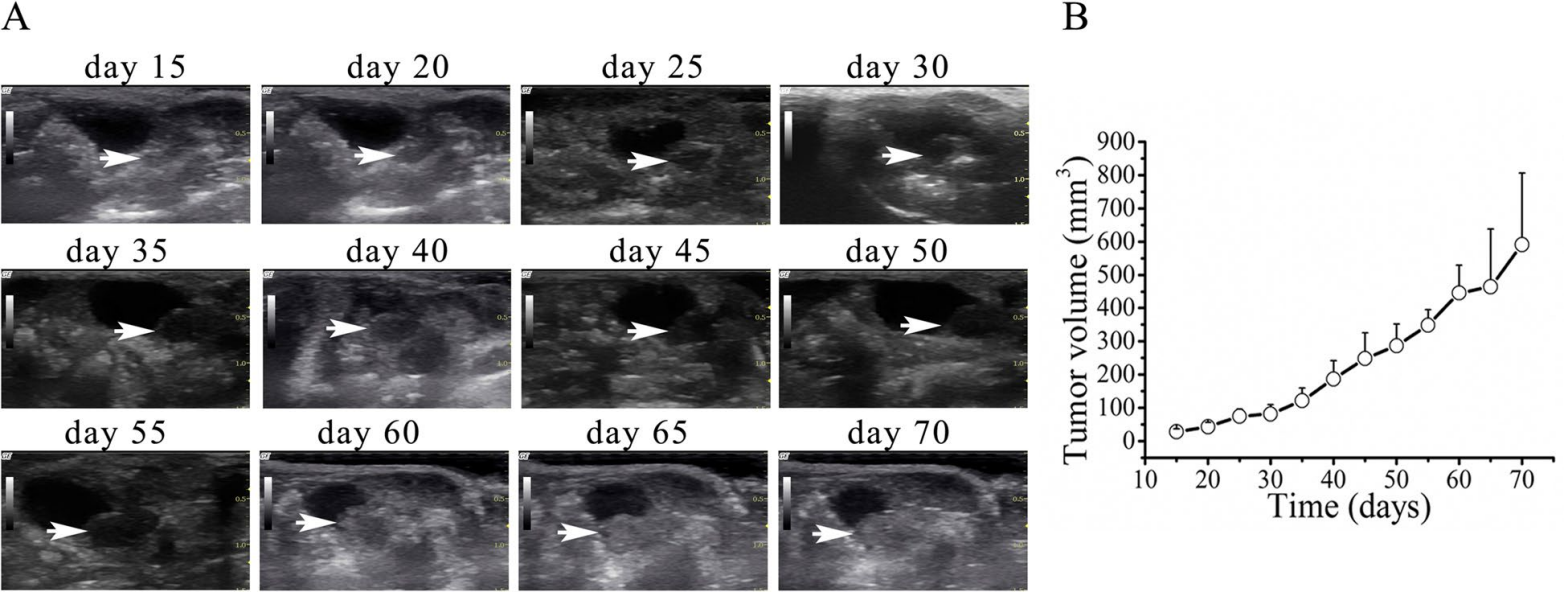
Gray scale ultrasound images showing orthotopic prostate tumor growth. **a** Day 15-Day 70: representative images in the transverse plane. The tumor (white arrow) progressed from a hypoechoic, well-defined, rounded structure underneath the bladder to an oval-shaped isoechoic structure that pushed the bladder anteriorly in the abdominal cavity. **b** Tumor growth curve. The circle represents the mean value of tumor volume, and the bar represents the standard deviation of tumor volume

On Day 35 and Day 70 after inoculation, mean orthotopic prostate tumor volume measured on gray-scale US were 122.5 ± 37.24 and 590.89 ± 214.95 mm^3^, respectively. After 8 weeks, the tumor take rate was 65% (*n* = 13/20 mice) (Fig. 3).

### T_2_-weighted MRI and correlation between tumor volume on US and MRI

Orthotopic prostate tumors were visualized on MRI on Day 35 and Day 70. At both timepoints, tumors could be easily distinguished from adjacent structures. As they grew, tumors maintained a compact and globe-like structure and frequently displayed increased intratumor signal heterogeneity.

**Fig. 4:**
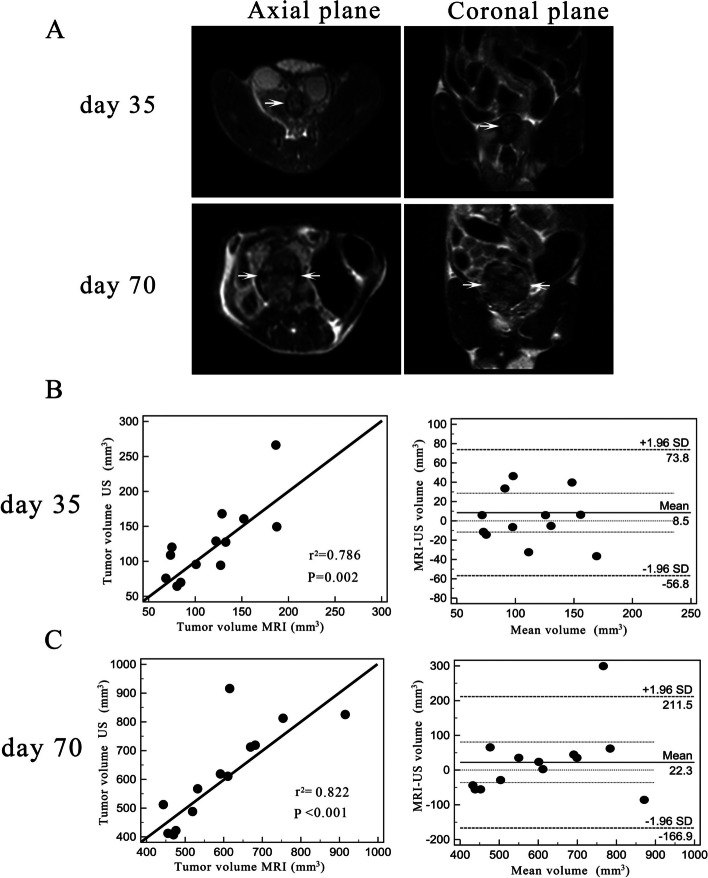
Orthotopic prostate tumor growth on MRI on Day 35 and Day 70 and the correlation between tumor volume measured on MRI and US. **a** On Day 35, the tumor was discernible by its position under the bladder and a T2W hyperintense signal. Linear correlation and Bland–Altman analysis of tumor volume measured on MRI and US on Day 35 (**b**) and Day 70 (**c**). The correlation between tumor volume measured by MRI and US was good. On the Bland–Altman plot, the majority of the differences were within ± two standard deviations of the mean. *r*^2^ = coefficient of determination, US = ultrasound, MRI = magnetic resonance imaging

On Day 35 and Day 70 after inoculation, mean orthotopic prostate tumor volume measured on MRI was 125.26 ± 53.87 and 617.03 ± 169.64 mm^3^, respectively. On Day 35 and Day 70, the correlation between tumor volume measured on MRI and gray-scale US was good at *r*^2^ = 0.786 and *r*^2^ = 0.822, respectively. In addition, the Bland–Altman plot shows that the volumes measured using US and MRI were consistent. These data indicate that both techniques have similar utility for monitoring orthotopic prostate tumor growth (Fig. 4).

### CEUS and data analysis

**Fig. 5:**
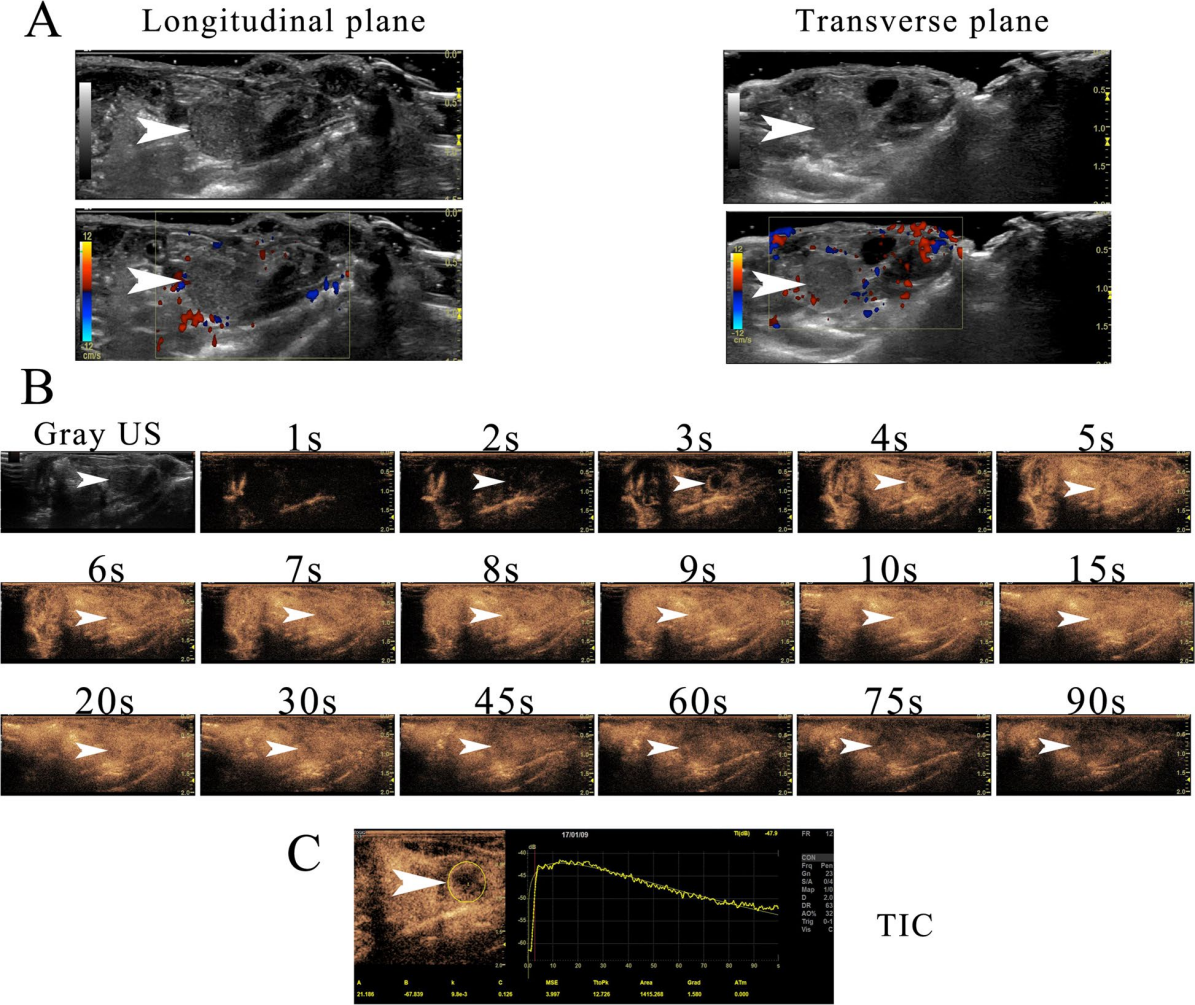
CEUS of an orthotopic prostate tumor. **a** B-mode and CDFI of a tumor in the longitudinal and transverse planes; CDFI showed no clear blood flow signal. **b** After SonoVue administration, the tumor margin enhanced more rapidly than the inner portion of tumor tissue in the early phase, with a well circumscribed rim appearing as a ring sign, and the central zone showing heterogeneous enhancement. **c** According to the TICs, signal intensity increased rapidly, peaked, and decreased gradually

Color Doppler flow imaging (CDFI) of orthotopic prostate tumors showed no blood flow signal. After SonoVue administration, the abdominal aorta enhanced rapidly, and the orthotopic tumor enhanced in the early phase, revealing a rich microcirculation. The tumor margin enhanced more rapidly than the inner portion of tumor tissue. A well circumscribed rim appeared as a ring sign at 3 s after SonoVue administration, the central zone of the tumor showed heterogeneous enhancement at 4 s, and the whole tumor was enhanced at 5 s. Signal intensity decayed steadily after peak enhancement. The tumor presented as dark tissue after 60 s. According to the TICs, signal intensity increased rapidly, peaked, and decreased gradually (Fig. 5).

### Histopathological characterization of orthotopic prostate tumors and serum PSA measurements

**Fig. 6:**
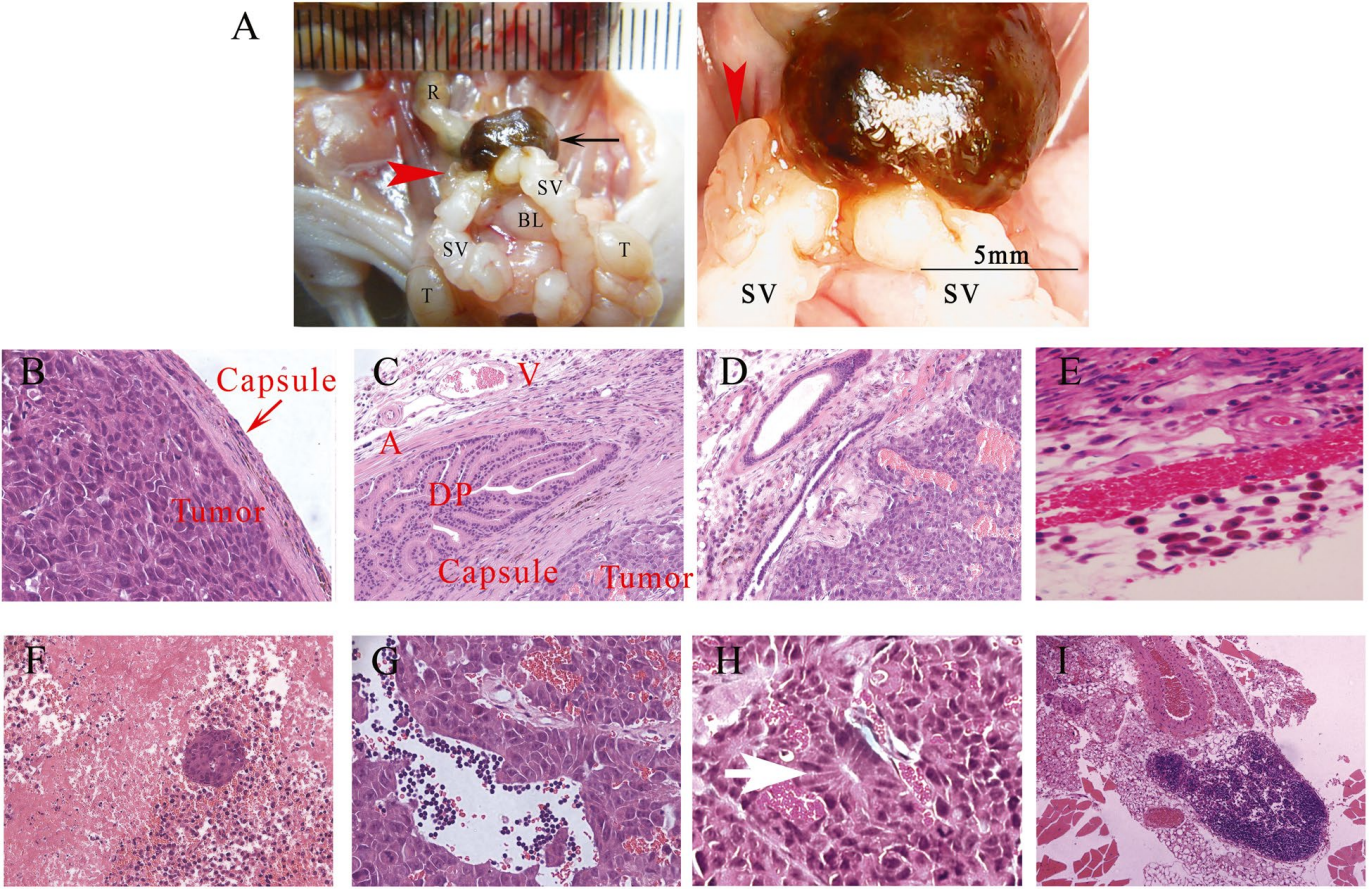
Macroscopic and microscopic characteristics of an orthotopic prostate tumor. **a** The tumor (black arrow) arose from the lateral aspect of the DP; the contralateral DP was unaffected (red arrow head). **b** The free border of the tumor was covered with a uniform fibrous capsule. Tumor cells showed pronounced cytologic atypia with uniformly enlarged nuclei. **c** Acini were entrapped by a variably fibrotic stroma. **d** Acini had sharp, angular contours. **e** A considerable peritumoral inflammatory infiltrate containing neutrophils and macrophages was present. **f** The intratumoral region displayed gross hemorrhage, substantial necrosis and remnant islands of tumor cells. **g** The intratumoral region was infiltrated by neutrophils. **h** Haphazardly arranged microacinar structures were lined by high columnar tumor cells (white arrow). **i** The peritumoral and regional lymph nodes showed pronounced inflammation. SV, Seminal vesicle; BL, bladder; R, rectum; T, testis; A, artery; V, vein; DP, dorsal prostate lobes; Magnification: **I** × 4, **C**, **D**, and **F** × 10, and **B**, **E**, **G**, and **H** × 20

Observations of gross specimens showed orthotopic prostate tumors were characterized by spherical expansion. Tumors were well circumscribed, round, dark brown, and soft, with a smooth outer surface and a glossy appearance. Tumors were confined to the unilateral DP, while the contralateral DP remained intact. Histologic findings showed the tumor-stroma interface was smooth with well-defined margins, often displacing or overrunning adjacent tissues and typically containing numerous macrophages. Tumors subtly encircled a native tubule, and the free border of the tumor was covered with a uniform 28.34 ± 2.74 μm thick fibrous capsule. Tumors were composed of anaplastic epithelial cells with pleomorphic nuclei and prominent nucleoli. Tumor cells were arranged in acini encircled by fibrous septa with variably thickened walls. Adjacent peritumoral ducts were closely packed into intersecting bundles of fibroblasts. The intratumor region displayed gross hemorrhage, substantial necrosis and cell death, and was infiltrated by abundant neutrophils. The enlarged external iliac lymph node presented an acute inflammatory reaction (Fig. 6). No metastases were found in the lung, liver, kidney, or adrenal glands. No bone metastases were found in the vertebrae or femur. PSA levels ranged from 3.7–219.0 ng/mL (mean 103.6 ± 68.1 ng/mL).

### Immunohistochemical characterization of orthotopic prostate tumors

**Fig. 7:**
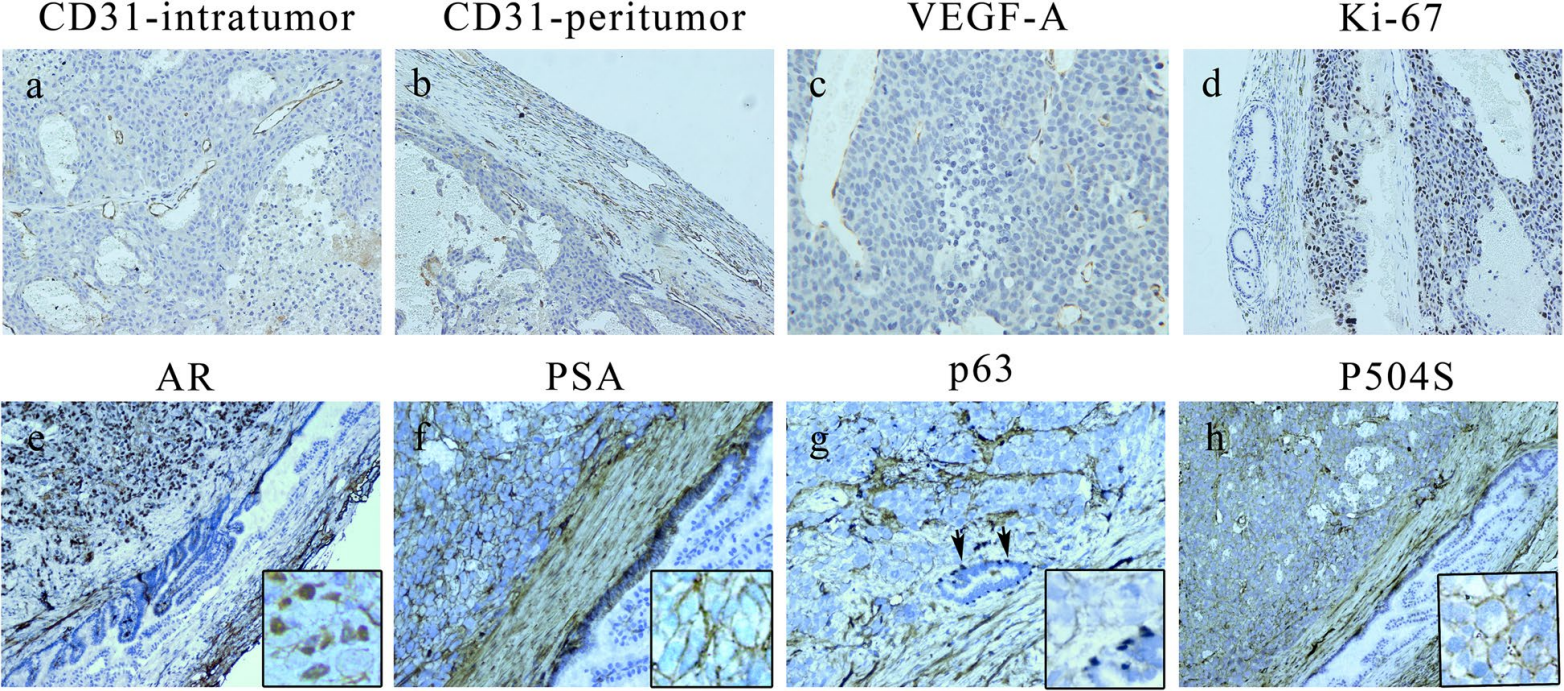
Immunohistochemistry of an orthotopic prostate tumor. **a** and **b** Tumors contained a rich network of vessels. Intratumoral vessel diameter was larger than peritumoral vessel diameter, but microvessel density was increased in the peritumoral region. **c** The VEGF-A protein was positively expressed. **d** Tumors were human-specific Ki-67 positive. **e** and **f** Tumor cells showed strong positive nuclear staining for human AR, and moderate positive cytoplasmic staining for human-specific PSA. **g** and **h** Tumor cells were p63 negative and showed diffuse P504S expression. The contralateral normal DP was diffusely positive for p63 in the basal cell layer and AR, PSA, and P504S negative. Magnification: **a**, **b**, and **c** × 4, **d** × 10, and **e–g** × 20

Immunohistochemical staining of orthotopic prostate tumors using CD31 antibodies specific for human endothelial cells revealed abundant blood capillaries. The tumor capsule and peritumoral area contained a rich network of vessels with a smaller diameter than intratumoral vessels. Tumors were VEGF-A and human-specific Ki-67 positive and p63 negative. Tumor cells were diffusely and weakly positive for P504S, showed strong positive nuclear staining for human AR, and moderate positive cytoplasmic staining for human-specific PSA. The contralateral normal DP was diffusely positive for p63 in the basal cell layer and AR, PSA, and P504S negative (Fig. 7).

## Discussion

Establishing a reliable and reproducible clinically relevant orthotopic LNCaP xenograft mouse model is challenging in tumor implantation and the tumorigenicity of LNCaP cells [10]. Firstly, the mouse and human prostates have similar embryological development, cellular composition, and molecular characteristics, but their anatomy and histology differ [11]. The mouse prostate is tiny, and the orthotopic injecting of tumor cell suspensions into the tiny mouse prostate remains a challenge. A number of orthotopic models have been established using subcutaneous tumor fragments (1 mm^3^) inserted into the mouse prostate [12, 13]. However, the subcutaneous and orthotopic microenvironment of tumor are definitely different. There is a significant difference in expression of hundreds of genes related to tumor biological progression between subcutaneous and orthotopic grown tumor [14]. Orthotopic models after tumor cell injection can better mimic the growth of human prostate tumor compared with subcutaneous models. Moreover, attention should be paid to the excision of the prostate capsule in mice, since the opened prostate capsule may facilitate tumor growth and metastasis [15–17]. Secondly, although PC-3 and Du145 had a high take rate, they are hormone-independent human prostate cancer cell line [18]. The LNCaP cell line is the only androgen-dependent human prostate cancer cell line that expresses AR and PSA, which can better reflect the growth of human prostate tumor. However, the inoculation of orthotopic tumors in the prostate of nude mice is technically challenging, and the tumorigenicity of LNCaP cells is low.

A comprehensive understanding of the anatomy and histology of the mouse prostate is essential when establishing a mouse model of prostate cancer. The mouse prostate is comprised of four functionally and morphologically distinct lobes that encircle the urethra [19, 20]. The AP, or coagulating gland, is the largest lobe. The AP is bilateral and attached to the lesser curvature of the SVs [21]. The DP and LP may be collectively referred to as the dorsolateral lobes [11]. The LP runs parallel to the urethra and has large diameter acini [22]. The VP is a single structure located ventrally in the midline above the urethra.

In the present study, LNCaP cells were inoculated into the unilateral DP of Balb/c athymic nude mice. The DP was the most suitable ectopic site for inoculation as gene expression patterns indicate that the mouse DP is similar to the peripheral zone of the human prostate, where the majority of human cancers arise [23]. In Balb/c athymic nude mice, the DP and LP were easily discriminated by a neurovascular bundle located on the posterolateral aspect of the prostate that ran parallel to the urethra and inserted obliquely into the dorsal aspect of the LP and the ventral aspect of the DP. Thus, although the DP had a deep location, orthotopic inoculation was relatively easy. The VP was difficult to visualize as the puboprostatic ligament, which appeared as a broad, strong ligament lying anterior to the VP and running medially in the sagittal orientation, pushed the VP deep to the urethra. The LP encircled the VD at the insertion site of the prostate between the base of the bladder and the root of the SV, a feature that deserves further anatomical investigation in future studies.

Inoculation of LNCaP cells into the DP of Balb/c athymic nude mice was performed under the guidance of a stereoscopic microscope. This required some methodological considerations. First, the SVs were gently lifted out of the abdominal cavity with forceps and moved posteriorly with a cotton ball to expose the DP. Microforceps were not used to expose the DP to avoid damaging the SVs [24], which are fragile and contain a milky, sticky secretion that could interfere with dissection. Second, although previous studies have externalized the bladder and SVs from the abdominal cavity [6], this may overstretch the pubourethral ligament and cause urinary incontinence; therefore, the bladder was retained in the abdominal cavity. Third, the SV was stabilized with a cotton ball to facilitate piercing the DP. This minimized leakage of the cell suspension, which is essential as orthotopic xenografts have very low take rates. Last, the volume and number of cells required to develop an orthotopic prostate tumor model vary across mouse strains and lobe of the prostate [25]. We recommend injecting 20μL suspension containing 1.0 × 10^6^ LNCaP cells into the unilateral DP of each animal when using Balb/c athymic nude mice.

The choice of ectopic site can affect tumor growth [26]. Orthotopic LNCaP xenografts grow more rapidly than subcutaneous LNCaP xenografts [5]. Orthotopic LNCaP xenografts grow larger in the mouse DP than the VP and LP due to anatomical differences. Tumors can freely expand around the periprostatic space in the pouch of Douglas beside the DP, proving the opportunity for optimal growth [27]. US is beneficial for imaging orthotopic prostate tumors. US can be used in real-time, is non-ionizing, and has good sensitivity and spatial resolution. US has utility for the detection of orthotopic tumors early after cell inoculation and can clearly delineate tumors from surrounding tissues to yield important information about the precise location of a tumor [28, 29]. MRI can help determine orthotopic prostate tumor volume [30], but MRI is not a cost-effective or convenient modality to use when routinely monitoring tumorigenesis in orthotopic models.

Accumulating evidence suggests that angiogenesis is involved in progression and metastasis in prostate cancer. In human prostate cancer, angiogenesis results in increased capillary density and changes in vascular morphology [31]. In the mouse prostate, tumorigenesis is accompanied by increased vascularization and vascular leakage [25, 32]. In the present study, CDFI showed no blood flow signal in orthotopic prostate tumors in Balb/c athymic nude mice; however, in accordance with previous reports, CEUS with SonoVue showed a high degree of neo-vascularization [28], with margins of orthotopic prostate tumors enhancing more rapidly and with higher signal intensity than tissue inside the tumors [33]. Consistent with this, immunohistochemical staining with CD31 antibodies specific for human endothelial cells revealed abundant blood capillaries in the orthotopic prostate tumor capsules and peritumoral regions. The expression of angiogenesis-related VEGF-A in orthotopic tumors is positive, which is in accordance with the results of CEUS. Thus, the DP of the Balb/c athymic nude mouse provided a suitable pro-angiogenic microenvironment.

Morphological observations of orthotopic prostate tumors in the present study revealed tumors resembled high-grade cancer, similar to Gleason Pattern 5. Tumors were sharply circumscribed and confined predominantly within a fibrous capsule. The interface between the tumor and the DP was demarcated, and the tumors subtly encircled a native tubule. Small distorted acini were entrapped by a variably fibrotic stroma. The presence of microacinar‐like structures represented a significant histology, mimicking human adenocarcinoma, which is the most predominate architectural pattern of human prostate cancer [34]. A considerable peritumoral inflammatory infiltrate was present, consistent with a previous study in an orthotopic prostate tumor rat model, which indicated increased tumor associated-macrophages correlated with a poor prognosis [35].

The majority of prostate cancers stain positive for AR. The AR signaling pathway is active during prostate cancer progression [36]. PSA has utility for identifying poorly differentiated prostate cancer. P504S expression is upregulated in prostate cancer, and p63 is a diagnostically useful basal cell marker in benign lesions. In the present study, orthotopic prostate tumors stained positive for AR, PSA, and P504S and were p63 negative. These data imply that the orthotopic LNCaP xenograft mouse model is an excellent tool to investigate the molecular mechanisms of prostate cancer progression [3].

## Conclusion

In conclusion, this study describes a successful approach to establishing an orthotopic LNCaP xenograft Balb/c athymic nude mouse model. The model requires a thorough understanding of the mouse prostate anatomy and proper technique. Tumor growth can be tracked by real-time US. Tumor pathology is similar to Gleason Pattern 5. CEUS vividly showed an angiogenic process, suggesting the DP provided a suitable prostatic microenvironment for angiogenesis. The orthotopic LNCaP xenograft Balb/c athymic nude mouse model represents a valuable tool for the in vivo study of the biological processes involved in angiogenesis in prostate cancer, as well as for preclinical evaluations of novel anti-angiogenic therapies.

## Data Availability

The datasets and analyzed during the current study are available from the corresponding author on reasonable request.

## Abbreviations

PSA: Prostate-specific antigen
PSMA: Prostate specific membrane antigen
AP: Anterior prostate
LP: Lateral prostate
VP: Ventral prostate
DP: Dorsal prostate
US: Ultrasound
CEUS: Contrast-enhanced ultrasonography
SVs: Seminal vesicles
ROI: Region of interest
TIC: Time-intensity curve
MRI: Magnetic resonance imaging
ECLIA: Electro-ChemiLuminescence ImmunoAssay
VEGF-A: Vascular endothelial growth factor A
AR: Androgen receptor
VD: Vas deferens
CDFI: Color Doppler flow imaging
