# Correction: Establishment of an orthotopic prostate cancer xenograft mouse model using microscope-guided orthotopic injection of LNCaP cells into the dorsal lobe of the mouse prostate

**DOI:** 10.1186/s12885-022-09310-z

**Published:** 2022-02-22

**Authors:** Weiyong Liu, Yunkai Zhu, Lei Ye, Yajuan Zhu, Yuhao Wang

**Affiliations:** 1 Department of Ultrasound, The First Affiliated Hospital of USTC, Division of Life Sciences and Medicine, University of Science and Technology of China, Hefei, 230001 Anhui China; 2 Department of Ultrasound, Xinhua Hospital Affiliated to Shanghai Jiaotong University, 200092 Shanghai, People’s Republic of China; 3 Department of Clinical Medicine, Wannan Medical College, Wuhu, 241002 Anhui People’s Republic of China

**Correction to: BMC Cancer 22, 173 (2022)**

**https://doi.org/10.1186/s12885-022-09266-0**

Following publication of the original article [1], the authors identified an error in affiliation 1.

The correct affiliation 1 is:

Department of Ultrasound, The First Affiliated Hospital of USTC, Division of Life Sciences and Medicine, University of Science and Technology of China, Hefei, Anhui, 230001, China
